# Jumbo quail responses to diets containing incremental levels of apple (*Malus domestica* Borkh) pomace

**DOI:** 10.1007/s11250-024-04240-3

**Published:** 2024-11-26

**Authors:** Allen Ngoanaoroele Matabane, Chidozie Freedom Egbu, Caven Mguvane Mnisi

**Affiliations:** 1 Department of Animal Science, School of Agricultural Science, North-West University, Mafikeng, South Africa; 2 Food Security and Safety Niche Area, Faculty of Natural and Agricultural Science, North-West University, Private Bag x2046, Mmabatho, 2735 South Africa

**Keywords:** Agro-industrial waste, Growth performance, Hemato-biochemistry, Nutraceutical, Product quality, Quail

## Abstract

The utilization of nutraceutical sources such as apple (*Malus domestica* Borkh) pomace powder (APP) could be a strategy to reduce the excessive disposal of this agro-waste in landfills and promote sustainable Jumbo quail (*Coturnix* sp.) production. However, the amount of the APP that can be included in Jumbo quail diets to achieve optimum production is unknown. This study evaluated the impact of including incremental levels of APP on performance metrics, haemato-biochemical parameters, and meat quality responses in Jumbo quail. A total of 350 Jumbo quail (7-day-old; 28.0 ± 0.817 g live weight) were weighed and randomly allotted to five dietary treatments, where each treatment had seven replicates with 10 birds each. The diets were formulated to contain 0, 25, 50, 75, and 100 g/kg APP. Dietary APP induced negative quadratic effects (*p* < 0.05) on feed intake in weeks 2 and 3. Body weight gain and gain-to-feed ratio decreased linearly in weeks 2, 3, and 4, but quadratically responded to APP levels in week 5. Platelets, heterophils, lymphocytes, and serum phosphorus and calcium showed linear or quadratic responses (*p* < 0.05) as APP levels increased. Increasing levels of APP linearly (*p* < 0.05) reduced carcass performance and 24-hour breast meat redness and chroma values but increased 1-hour yellowness and 24-hour lightness. The inclusion of APP compromised growth and carcass performance in young growing Jumbo quail. However, feed efficiency and final body weight were maximized between 50 and 75 g APP levels/kg diet in five-week-old Jumbo quail without compromising blood and meat quality parameters.

## Introduction

Emerging bird species such as the Jumbo quail (*Coturnix* sp.) are steadily introduced in conventional poultry production to increase the supply of poultry products in the agro-food value chain (Henchion et al. 2021; Hlatshwayo et al. 2023). The Jumbo quail is the largest *Coturnix* quail breed that is currently reared for meat production (Marareni and Mnisi 2020). Due to their low input requirement, sustainable quail production has great potential to alleviate hunger in poor communities while contributing to household nutrition and food security (Masenya et al. 2021). Quail are highly adaptable and resilient birds known for their rapid growth rates, low bone-to-meat ratio, low mortality rates, and high feed efficiency, among others (Shehata et al. 2021; Ustundag and Ozdogan 2023). However, the growth of the quail farming business in South Africa is threatened by rising feed costs, which are influenced by the over-reliance on highly demanded feed ingredients (maize and soybean grains) used to formulate commercial poultry diets.

Thus, agro-waste by-products with functional bioactivities can be incorporated into Jumbo quail diets to promote economic viability and environmental sustainability in animal agriculture. Apple (*Malus domestica*) pomace (AP), consisting of peels, pulp, seeds, and stems, is one such agro-waste that is generated from apple processing. South Africa produced 1,231, 866.87 tons of apples in 2022, generating 307, 966.72 tons of AP in 2022 (FAOSTAT 2024). The AP is traditionally discarded through incineration or landfilled to the detriment of the environment, near apple processing factories (Gowman et al. 2019; Fernandez et al. 2023). Landfilling of AP leads to an increase in methane production, contamination of groundwater, loss of biodiversity, and soil degradation (Erinle and Adewole 2022; Gołębiewska et al. 2022). Therefore, the utilization of APP in quail diets could provide a long-term upcycling strategy to manage its disposal in landfills. Moreover, AP has a high nutraceutical value that is characterized by high levels of simple carbohydrates, triglycerides, fatty acids, vitamin C, potassium, calcium, magnesium, flavonoids, phenolic acids, and procyanidins (Barreira et al. 2019; Lyu et al. 2020), making it an ideal candidate for use in Jumbo quail diets.

However, its utility as a feed ingredient may be limited by its low protein content and poor digestibility due to high levels of fibre and lignin-to-cellulose ratio (Patocka et al. 2020; Pollini et al. 2021). This indicates that higher APP levels could jeopardize Jumbo quail’s health and productive performance (Aghili et al. 2019). Unfortunately, no study has determined the optimum inclusion level of APP to ensure safe utilization in Jumbo quail. This study, therefore, assessed the effect of including incremental levels of APP on productive performance, physiological parameters, and qualitative meat characteristics in Jumbo quail.

## Materials and methods

### Animal rights statement

The methods for rearing and slaughter of the quail were reviewed and accepted (NWU-00807-21-A5) by North-West University’s Animal Production Research Ethics Committee.

### Ingredient sources, analyses, and diet formulation

Fresh, wet *M. domestica* (containing a mixture of yellowish, red, and green apples) was supplied by Elgin Fruit Juices (Pty) Ltd (Grabouw, South Africa). The AP was sun-dried for a week, during which it was regularly turned upside down to avoid fungal infestation and thereafter milled (2 mm) into powder. The APP was then analyzed for dry matter (DM), organic matter (OM), ash, crude protein (CP), ether extract (EE), and amino acids (threonine, lysine, tryptophan, and methionine) using methods described by the Association of Official Analytical Chemists (2005). Minerals (Ca, K, P, Na and Cl) were determined according to the Agri-Laboratory Association of Southern Africa methods (1998). The metabolizable energy content was calculated as described by Masenya et al. (2021). The Folin-Ciocalteau method (Makkar 2003) was utilized to determine the concentration of total soluble phenolics (TSP), which were expressed as tannic acid equivalents (g TAE/kg). The soluble condensed tannins (SCT) were analyzed using a mixture of 95% butanol and 5% HCl (Porter et al. 1986) and reported as absorbance units (AU) per 200 mg sample. The composition of the APP, as presented in Table 1, was then used to formulate five isocaloric and isonitrogenous diets to include 0 (AP0), 25 (AP25), 50 (AP50), 75 (AP75), and 100 g/kg APP (AP100) in a standardized conventional grower diet following the recommendations by Smith (2017). The APP has high levels of fiber and lignin-to-cellulose ratio (Patocka et al. 2020; Pollini et al. 2021), thus lower levels of up to 100 g/kg were used in this study. The experimental diets were also analyzed as described for the APP.

**Table 1:** Ingredient and nutritional composition (g/kg, as is basis) of diets containing incremental levels of apple pomace powder

^‡^Ingredients	APP	^†^Diets				
		AP0	AP25	AP50	AP75	AP100
Apple pomace powder		0.000	25.00	50.00	75.00	100.0
Yellow maize (8.5%)		437.3	524.3	469.3	334.0	440.8
Extruded full-fat soya		0.000	0.000	260.0	250.0	250.0
Soya oil cake (44%)		350.6	379.2	106.6	22.60	163.1
Sunflower oil cake (38%)		68.40	0.000	81.20	243.3	14.70
DL-Methionine		1.400	1.400	0.900	0.000	0.600
Soya oil crude		65.10	30.40	1.600	45.60	0.000
Limestone (slow solubility)		0.000	24.10	23.30	22.40	23.70
Salt (Fine)		1.600	1.700	1.700	1.500	1.800
Sodium bicarbonate		15.40	10.90	2.400	2.500	2.200
Premix		3.100	3.000	3.000	3.100	3.100
MDCP		56.10	0.000	0.000	0.000	0.000
Lysine-HCl		1.000	0.000	0.000	0.000	0.000
**Nutritional composition**						
Dry matter	952.0	922.1	941.6	935.6	944.2	967.8
Organic matter	885.2	861.0	890.3	882.8	887.0	907.4
Lysine	3.400	12.60	12.60	12.72	13.00	14.56
Methionine	1.200	5.000	5.000	5.000	5.000	5.000
Threonine	2.600	8.490	8.880	9.390	10.20	10.36
Crude protein	90.00	230.0	230.0	230.0	230.0	230.0
Crude fiber	29.00	37.46	39.67	41.75	43.86	44.50
Ether extract	50.00	87.89	65.83	71.54	108.0	75.77
ME (MJ/kg)	11.70	12.50	12.50	12.50	12.50	12.50
Calcium	1.000	10.10	10.10	10.00	10.00	10.00
Chloride	0.600	1.400	1.450	1.450	1.450	1.450
Potassium	3.300	9.520	9.640	8.790	8.220	9.000
Sodium	2.800	5.000	3.810	1.500	1.500	1.500
Total phosphorus	1.400	4.180	3.540	4.230	5.260	4.410
TSPh (g TAE/kg)	29.44	19.36	22.38	23.73	25.38	26.41
SCT (AU)	0.693	0.063	0.077	0.169	0.392	0.583

^†^Diets: AP0 = a conventional grower diet without apple pomace powder, AP25 = a conventional grower diet with 25 g/kg of apple pomace powder, AP50 = a conventional grower diet with 50 g/kg of apple pomace powder, AP75 = a conventional grower diet with 75 g/kg of apple pomace powder and AP100 = a conventional grower diet with 100 g/kg of apple pomace powder

^‡^Premix = copper sulphate 8.0 mg; ferrous sulphate 80 mg; zinc sulphate 79 mg; niacin 30 mg; magnesium sulphate 100 mg; vitamin A 11,000 IU; potassium iodide 0.34 mg; folic acid 0.7 mg; pantothenic acid 10 mg; biotin 0.12 g; vitamin B1 2.5 mg; vitamin B6 5.1 mg; vitamin B2 4.5 mg; vitamin E 25 IU; vitamin D3 2,500 IU; vitamin K3 2.0 mg; and sodium selenite 0.25 mg, MDCP = mono-dicalcium phosphate, ME = metabolizable energy, APP = apple pomace powder, TSPh = total soluble phenolics, SCT = soluble condensed tannins

### Feeding trial and experimental design

The experiment was carried out during summer at North-West University Experimental Farm (25°40.459′ S; 26°10.563′ E), where temperatures ranged between 25 and 37 °C and an average rainfall of approximately 450 mm. A total of 350, 4-day-old unsexed Jumbo quail chicks were bought from a quail breeder in Gauteng province, South Africa. For the first 3 days, all quail birds were offered a stress pack (containing a wide range of electrolytes and vitamins) administered via drinking water. The birds were acclimated to the diets and cages until 7 days of age. At day 7 of age, the birds were then weighed and randomly allocated into 35 cages (experimental units placed in the same quail house), such that each dietary group had 7 replicate cages. Each cage (100 cm L × 30 cm H × 60 cm W) carried 10 birds and the floors were covered with removable polythene plastics. The experiment was conducted from 7 to 35 days of age under natural lighting from morning (06h00) to evening (18h00) and the birds were slaughtered before exhibiting anatomical and physiological gender differences. The house temperature ranged between 27 and 33 °C, with relative humidity of 60%. Clean tap water and dietary treatments were provided *ad libitum* throughout the feeding trial.

### Growth performance

At 7 days of age, initial body weights (28.0 ± 0.817 g) were recorded, and live weights were subsequently recorded weekly per cage until day 35 of age to obtain average weekly body weight gain (BWG). Feed intake (FI) was calculated as the difference between the weight of feed offered and feed refused. Gain-to-feed ratio (G: F) was calculated as BWG divided by FI.

### Slaughter procedure and blood analysis

Final body weight (FBW) was determined by weighing all quail birds on day 35 and thereafter slaughtered at a nearby abattoir by exsanguination. About 4 ml of fresh blood samples were collected during bleeding from two random birds per experimental unit into hematological and serum tubes (5 ml). Whole blood samples were analyzed within 24 h of collection (Washington and Van Hoosier 2012) to determine the concentration of hematocrits, platelets, heterophils, lymphocytes, monocytes, and white cell count (WCC) using an automated LaserCyte Hematology Analyzer (IDEXX Laboratories Pty Ltd, Johannesburg, South Africa). Upon centrifuging, serum biochemical parameters (albumin, total protein, creatinine, amylase, globulin, calcium, alanine transaminase (ALT), phosphorus, choline, lipase, albumin/globulin ratio (ALB/GLOB), symmetric dimethylarginine (SDMA), total bilirubin (TBIL), and alkaline phosphatase (ALKP) was determined using an automated Vet Test Chemistry Analyzer (IDEXX Laboratories Pty Ltd, Johannesburg, South Africa).

### Carcass features, internal organs, and meat quality

The carcasses were thereafter de-feathered, eviscerated, and immediately weighed to obtain hot carcass weight (HCW) and re-weighed after chilling (4 °C) for 24 h to measure cold carcass weight (CCW). Back length (cm) as well as carcass cuts (thigh, drumstick, breast, and wing) and internal organs (proventriculus, liver, spleen, gizzard, small intestine, caecum, and colon), were weighed and expressed as a proportion of CCW (g / 100 g CCW). Dressing % was determined as a proportion of HCW on FBW.

A digital pH meter fitted with a spear-type electrode was used to measure the 1 h and 24 h pH of breast meat (Henchion et al. 2021; Masenya et al. 2021). Breast meat color coordinates (L* = lightness, b* = yellowness, and a* = redness) were determined using a colorimeter (CIE 1978). The calculations of hue angle and chroma values were done using the formulae by Priolo et al. (2002). Breast meat water holding capacity (WHC) was measured as guided by Pouttu and Puolanne (2004). Shear force was determined following the methods by Lee et al. (2008) using a Meullenet-Owens razor shear blade mounted on a Texture Analyzer (TA XT plus, Stable Micro Systems, Surrey, UK). The raw samples were then weighed and cooked until a core temperature of 75 °C for 15 min to determine cook losses (Honikel 1998).

### Statistical analyses

All measured responses were subjected to normality tests using the normal option in the Proc Univariate statement (SAS version 9.4 2013). The data were further evaluated for linear and quadratic effects using the procedure of response surface regression (SAS version 9.4 2013). The optimum inclusion level of APP was determined using the non-linear equation: *y = ax*^*2*^ *+ bx + c*, where: *y* is the dependent response; *a* and *b* are the coefficients of the quadratic equation; and *c* is the intercept. The *x* value for an optimal response was calculated as $\:\frac{-b}{2a}$. The level of significance was proclaimed at *p* < 0.05.

## Results

### Growth performance

Table 2 indicates that increasing APP levels induced positive linear and negative quadratic effects on feed intake in week 2 [R^2^ = 0.515; *p* = 0.015]. In week 3, incremental levels of APP resulted in a negative quadratic effect for feed intake [R^2^ = 0.160; *p* = 0.024], which was maximized at 42.5 g/kg APP level. Linear decreases were recorded for weight gain in week 2 [R^2^ = 0.567; *p* = 0.0001], week 3 [R^2^ = 0.528; *p* = 0.0001] and week 4 [R^2^ = 0.527; *p* = 0.0001]. In week 5, a negative quadratic response was recorded for weight gain [*y* = 34.60 (± 2.890) + 0.33 (± 0.137) *x* – 0.003 (± 0.005) *x*^2^; R^2^ = 0.156; *p* = 0.046], which was maximized at 55.2 g APP /kg diet. Similarly, linear decreases were recorded for G: F in week 2 [R^2^ = 0.799; *p* = 0.0001], week 3 [R^2^ = 0.609; *p* = 0.0001] and week 4 [R^2^ = 0.649; *p* = 0.0001]. Likewise, a negative quadratic response was observed for G: F in five-week-old quail [*y* = 0.240 (± 0.017) + 0.002 (± 0.001) *x* – 0.00002 (± 0.00001) *x*^2^; R^2^ = 0.151; *p* = 0.024], which was maximized at 50 g APP /kg diet according to the equation.

**Table 2:** Growth performance of jumbo quail fed diets containing incremental levels of apple pomace powder

	^†^Diets					^‡^SEM	Significance	
	AP0	AP25	AP50	AP75	AP100		Linear	Quadratic
Feed intake (g/bird)								
7–14 d	63.46	71.52	71.93	76.27	74.57	1.61	0.0001	0.010
15–21 d	96.94	103.1	96.38	106.7	92.16	2.26	0.501	0.024
22–28 d	130.5	122.6	127.9	133.8	123.0	2.03	0.650	0.594
29–35 d	148.7	141.7	151.9	161.9	149.5	2.68	0.051	0.396
Weight gain (g/bird)								
7–14 d	29.64	30.41	26.02	24.93	22.64	0.92	0.0001	0.437
15–21 d	43.34	39.91	37.17	39.07	31.47	1.15	0.0001	0.454
22–28 d	49.63	40.25	41.45	39.74	34.98	1.43	0.0001	0.299
29–35 d	35.36	40.61	39.74	46.84	34.39	2.94	0.661	0.023
Gain-to-feed ratio (g: g)								
7–14 d	0.467	0.428	0.362	0.326	0.304	0.01	0.0001	0.163
15–21 d	0.447	0.388	0.387	0.366	0.341	0.01	0.0001	0.218
22–28 d	0.381	0.328	0.324	0.297	0.284	0.01	0.0001	0.125
29–35 d	0.238	0.286	0.262	0.288	0.230	0.02	0.824	0.024

^†^Diets: AP0 = a conventional grower diet with no apple pomace powder inclusion, AP25 = a conventional grower diet with 25 g/kg of apple pomace powder, AP50 = a conventional grower diet with 50 g/kg of apple pomace powder, AP75 = a conventional grower diet with 75 g/kg of apple pomace powder, and AP100 = a conventional grower diet with 100 g/kg of apple pomace powder

^‡^SEM: standard error of the mean

### Blood parameters

Platelet count linearly increased [R^2^ = 0.180; *p* = 0.017] as APP levels increased (Table 3). Linear improvements were recorded for lymphocytes [R^2^ = 0.303; *p* = 0.001], and phosphorus [R^2^ = 0.188; *p* = 0.018] whereas a decrease in calcium was observed as APP level increased [R^2^ = 0.188; *p* = 0.026].

**Table 3:** Hematological and serum biochemical parameters of jumbo quail fed diets containing incremental levels of apple pomace powder

^‡^Parameters	^†^Diets					^§^SEM	Significance	
	AP0	AP25	AP50	AP75	AP100		Linear	Quadratic
Hematocrits (%)	43.53	41.86	45.71	46.24	47.07	1.53	0.089	0.529
^2^WCC (×10^9^/L)	21.67	21.54	22.53	22.77	23.63	2.28	0.543	0.875
Platelets (×10^9^/L)	22.63	18.73	27.91	27.82	34.74	4.21	0.017	0.434
Heterophils (%)	49.91	37.89	39.93	41.74	43.09	4.18	0.124	0.564
Lymphocytes (%)	35.71	40.06	42.97	44.87	53.79	3.60	0.001	0.541
Monocytes (×10^9^/L)	1.030	1.140	1.460	1.690	1.810	0.41	0.766	0.163
SDMA (µg/dL)	30.00	35.29	23.73	30.14	35.00	4.39	0.734	0.316
Phosphorus (mmol/L)	3.590	4.480	4.640	4.660	4.910	0.36	0.018	0.293
Creatinine (µmol/L)	9.000	8.560	8.960	11.41	8.640	1.17	0.576	0.562
Calcium (mmol/L)	2.010	2.240	2.080	1.830	1.830	0.10	0.026	0.170
Total protein (g/L)	31.21	31.43	32.00	33.29	35.79	1.87	0.503	0.445
Albumin (g/L)	15.14	14.36	15.79	14.14	13.36	0.82	0.155	0.325
Globulin (g/L)	21.43	21.57	21.93	21.57	22.14	1.63	0.776	0.981
ALB/GLOB	0.740	0.790	1.000	0.730	0.720	0.13	0.839	0.236
ALT (U/L)	31.86	37.93	32.29	39.29	42.21	2.91	0.092	0.123
ALKP (U/L)	166.1	175.4	234.0	261.4	320.4	23.3	0.924	0.062
TBIL (µmol/L)	2.430	3.000	2.390	2.990	2.500	0.31	0.899	0.454
Choline (mmol/L)	3.030	3.860	2.810	2.710	2.700	0.27	0.065	0.540
Amylase (U/L)	138.8	214.9	164.6	180.9	199.4	20.3	0.215	0.556
Lipase (U/L)	115.6	170.1	186.8	125.9	191.7	19.2	0.120	0.497

^†^Diets: AP0 = a conventional grower diet with no apple pomace powder inclusion, AP25 = a conventional grower diet with 25 g/kg of apple pomace powder, AP50 = a conventional grower diet with 50 g/kg of apple pomace powder, AP75 = a conventional grower diet with 75 g/kg of apple pomace powder, and AP100 = a conventional grower diet with 100 g/kg of apple pomace powder

^‡^Parameters: WCC = white cell count, SDMA = serum symmetric dimethylarginine, ALB/GLOB = albumin/globulin ratio, ALT = alanine transaminase, ALKP = alkaline phosphatase, TBIL = total bilirubin

^§^SEM: standard error of the mean

### Carcass characteristics and visceral organ sizes

Table 4 indicates that there were linear decreases for HCW [R^2^ = 0.574; *p* = 0.0001], CCW [R^2^ = 0.577; *p* = 0.0001] and FBW [R^2^ = 0.514; *p* = 0.0001] in response to APP levels. Furthermore, APP levels caused a linear reduction in drumstick [R^2^ = 0.389; *p* = 0.0001], thigh [R^2^ = 0.283; *p* = 0.002] and breast weights [R^2^ = 0.432; *p* = 0.0001]. Small intestine weight [R^2^ = 0.139; *p* = 0.046] linearly increased with increasing APP levels. However, proventriculus weight showed a negative linear and positive quadratic effect [R^2^ = 0.319; *p* = 0.017] with increasing APP levels.

**Table 4:** Carcass characteristics and visceral organ sizes (%CCW, unless stated otherwise) of jumbo quail fed diets containing incremental levels of apple pomace powder

	^†^Diets					^§^SEM	Significance	
^‡^Parameters	AP0	AP25	AP50	AP75	AP100		Linear	Quadratic
FBW (g)	185.9	179.2	174.1	180.3	155.9	2.73	0.0001	0.065
Dressing (%)	65.74	62.77	63.80	60.44	62.73	1.62	0.111	0.318
HCW (g)	122.2	112.4	111.0	109.0	97.36	2.43	0.0001	0.574
CCW (g)	100.9	98.16	90.95	88.34	79.45	2.59	0.0001	0.426
Drumstick	2.965	2.810	2.654	2.729	2.444	0.08	0.0001	0.924
Thigh	4.405	3.887	4.142	3.976	3.393	0.17	0.002	0.413
Wing	3.388	3.461	3.242	3.596	3.002	0.22	0.361	0.355
Back (cm)	9.551	9.247	9.156	9.328	9.357	0.45	0.826	0.575
Breast	11.90	10.93	10.43	10.10	09.01	0.43	0.0001	0.967
Proventriculus	0.778	0.608	0.632	0.677	0.621	0.02	0.006	0.017
Gizzard	3.234	2.853	2.973	3.098	3.050	0.09	0.689	0.072
Liver	3.030	3.063	2.995	3.032	2.970	0.11	0.648	0.831
Spleen	0.188	0.208	0.201	0.249	0.196	0.02	0.316	0.172
Small intestine	5.684	6.108	6.005	6.300	6.204	0.19	0.046	0.366
Caecum	1.082	1.097	1.059	1.586	1.437	1.15	0.209	0.680

^†^Diets: AP0 = a conventional grower diet with no apple pomace powder inclusion, AP25 = a conventional grower diet with 25 g/kg of apple pomace powder, AP50 = a conventional grower diet with 50 g/kg of apple pomace powder, AP75 = a conventional grower diet with 75 g/kg of apple pomace powder, and AP100 = a conventional grower diet with 100 g/kg of apple pomace powder

^‡^Parameters: HCW = hot carcass weight, CCW = cold carcass weight, FBW = final body weight

^§^SEM: standard error of the mean

### Meat quality parameters

Linear increases were recorded for breast meat 1-hour yellowness (b*) [R^2^ = 0.156; *p* = 0.028] and 24-hour lightness (L*) [R^2^ = 0.130; *p* = 0.050] due to APP levels (Table 5). However, breast meat 24-hour redness (a*) [R^2^ = 0.251; *p* = 0.005] and 24-hour chroma [R^2^ = 0.282; *p* = 0.002] linearly declined with APP levels.

**Table 5:** Meat quality parameters measured 1-hour and 24-hours post slaughter in jumbo quail fed diets containing incremental levels of apple pomace powder

	^†^Diets					^§^SEM	Significance	
^‡^Parameters	AP0	AP25	AP50	AP75	AP100		Linear	Quadratic
pH_1_	5.954	5.959	5.836	5.996	5.949	0.05	0.908	0.376
pH_24_	5.119	5.587	5.010	5.241	5.053	0.10	0.227	0.279
L*_1_	48.99	48.79	48.99	48.36	48.81	1.05	0.805	0.900
L*_24_	40.86	44.00	39.68	40.05	39.14	1.06	0.050	0.434
a*_1_	6.077	5.454	5.402	5.161	6.238	0.58	0.987	0.141
a*_24_	5.641	5.520	4.760	5.160	4.910	0.18	0.005	0.209
b*_1_	2.909	3.007	2.938	2.147	2.129	0.34	0.028	0.448
b*_24_	3.397	3.557	2.997	2.935	2.826	0.36	0.121	0.975
Chroma_1_	6.759	6.301	6.216	5.667	6.712	0.55	0.674	0.218
Chroma_24_	6.620	6.630	5.700	6.010	5.710	0.21	0.002	0.458
Hue angle_1_	0.443	0.495	0.499	0.396	0.386	0.08	0.254	0.294
Hue angle_24_	0.537	0.574	0.553	0.508	0.516	0.06	0.538	0.696
WHC (%)	90.76	87.70	91.57	86.31	89.01	1.37	0.309	0.675
Drip loss (%)	4.697	4.857	3.898	3.908	4.556	0.44	0.385	0.248
CKL (%)	39.67	43.71	39.25	41.35	42.90	2.99	0.666	0.888
Shear force (N)	3.022	2.944	3.364	3.274	3.303	0.27	0.301	0.771

^†^Diets: AP0 = a conventional grower diet with no apple pomace powder inclusion, AP25 = a conventional grower diet with 25 g/kg of apple pomace powder, AP50 = a conventional grower diet with 50 g/kg of apple pomace powder, AP75 = a conventional grower diet with 75 g/kg of apple pomace powder, and AP100 = a conventional grower diet with 100 g/kg of apple pomace powder

^‡^Parameters: WHC = water holding capacity, L* = lightness, a* = redness, b* = yellowness, CKL = cooking loss

^§^SEM: standard error of the mean

## Discussion

The utilisation of nutraceutical sources such as apple pomace powder (APP) could be a strategy to reduce agro-waste disposal in landfills and promote sustainable production of emerging quail species such as the Jumbo quail. As anticipated, the analyzed nutritional composition of the APP-containing diets indicated increased crude fiber, TSPh, and SCT as the APP levels increased. The results showed that the ability of the birds to utilize and efficiently convert feed into body mass varied with age. Indeed, at 14 days of age, compensatory feed intake was seen from the young quail, which could have been a coping mechanism to deal with extra dietary fibre from the APP. Similarly, a negative quadratic response was noted for feed intake on days 21 and 28, maximized at a 42.5 g/kg APP level. This suggests an initial improvement in feed intake with increasing levels of APP up to 42.5 g/kg, after which the feed intake declined. This pattern can be explained by the gut-fill effect of dietary fiber and impaired nutrient absorption due to the increased passage rate through the digestive tract. Including APP up to 100 g/kg caused linear reductions in weight gain and feed efficiency from day 14–28 of age, confirming that the observed increment in feed consumption on day 14 was to compensate for nutrient deficiency due to high fibre in the APP-containing diets. Furthermore, the observed negative quadratic responses for BWG and G: F during days 29–35 could be attributed to the ability of the birds at this age to utilize APP’s phytochemicals and fiber levels. These results highlight the importance of determining optimum inclusion levels for alternative feed ingredients to maximize performance without compromising productive traits in Jumbo quail. High dietary fibre levels have been reported to increase the proportion of indigestible constituents in diets, which triggers compensatory feed intake (Colombino et al. 2020; Hlatshwayo et al. 2023). Consequently, this reduces feed utilization efficiency in birds due to gut restrictions (Hlatshwayo et al. 2023). The observed results support the finding by Matoo et al. (2001), who reported a reduction in BWG of broiler chicks supplemented with up to 50 g/kg dried APP. Likewise, Heidarisafar et al. (2016) observed a reduction in body weight of broilers at 42 days of age fed with diets containing 100 g/kg of dried APP, while Aghili et al. (2019) reported poor FCR in two-week-old broiler chicks supplemented with 120 g APP extract /kg of feed. Nonetheless, at five weeks of age, the birds demonstrated better performance which could have been facilitated by the development of the gastrointestinal tract as the birds grew older. In similar studies, Ayhan et al. (2009) and Colombino et al. (2020) reported an increase in weight gain of six-week-old and five-week-old broiler chickens reared on diets containing up to 100 g/kg and 150 g/kg dried APP, respectively. The improvement in weight gain indicates that matured birds efficiently utilized the diets thereby benefiting from polyphenolic bioactive compounds that have growth-boosting activities (Fermoso et al. 2018; Mourtzinos and Goula 2019).

Blood indices are commonly used to diagnose clinical diseases and monitor the effects of therapeutic, nutritional, and environmental factors (Peres et al. 2015). They are also used to monitor the pathophysiology and health status of animals, especially in cases of poor nutrition, chronic pathology, or stress conditions that do not show any clinical symptoms (Casanovas et al. 2021). In this study, platelet counts, which measure the amount of colourless blood cells that aid in blood clotting (Soslau 2020), linearly increased with APP inclusion. This could imply that APP polyphenols have hematopoietic properties. Hence, these phytochemicals stimulate the bone marrow to produce more platelets by either acting on the progenitor cells or by modulating the cytokine environment that supports thrombopoiesis. Furthermore, a linear increase was observed for lymphocytes indicating the ability of the quail to produce antibodies for protection (Safavipour et al. 2022). Lymphocytes are small white blood cells that play an influential role in defending the body against diseases by destroying pathogenic bacteria and viruses that invade the body (Chuang et al. 2007). Thus, the improvement in platelet and lymphocyte counts implies that the APP nutraceuticals had a beneficial effect on the health status of the birds.

In biochemical tests, the amount of calcium is important for structural development and egg production while serum phosphorus is needed for the growth, maintenance, and repair of all tissues and cells (Sahin et al. 2007). The observed linear increases in serum calcium and phosphorus indicate a mechanism of antioxidant and anti-peroxide activity of APP (Ghazalah et al. 2011). Apple pomace’s antioxidant compounds may help maintain proper calcium and phosphorus balance by protecting them from oxidative damage (Aghili et al. 2019). There were no dietary impacts on liver enzymes (ALT and ALKP), which indicates that APP inclusion does not cause liver damage. Most of the measured blood parameters were within the normal physiological range for healthy quail (Bolacali et al. 2021; Fikry et al. 2021). This implies that the inclusion of APP had no negative effect on the health and nutritional status of the birds.

The carcass weight and yield measurement is critical in the meat industry, as these factors are used to assess meat products and their market value (Egbu et al. 2022). The inclusion of APP linearly reduced carcass performance parameters. Cold carcass yield is believed to be an excellent predictor of total edible meat during storage, which implies that the inclusion of APP reduces the amount of edible meat in Jumbo quail. These results corroborate those of Habibian et al. (2016) and Zeferino et al. (2016), who reported that nutritional imbalances, nutrient shortages, or incorrect dilution of nutrients may have a negative effect on quail carcass quality. Thus, it is imperative to valorize APP prior to inclusion in poultry diets to ameliorate the antinutritional activities of secondary metabolites.

Internal organ sizes indicate the birds’ digestive health and function (Dlamini et al. 2023). Small intestine weight increased linearly with increasing APP levels. The enlargement of the small intestine could have been an anatomical and physiological mechanism for utilizing high dietary fibre in the APP groups (Oliveira et al. 2008; Laudadio et al. 2012). The proventriculus, which is a glandular organ in birds that aids in the initial chemical breakdown of feed before transiting to the gizzard for further digestion, showed a linear decrease with increasing APP levels. This could explain the observed poor feed efficiency in this study. Surprisingly, we did not observe any linear or quadratic responses for gizzard sizes despite the higher crude fiber content of the APP diets when compared to the control diets. This implies that 100 g/kg APP was insufficient to induce physio-anatomical changes of the gizzard. Similarly, Hlatshwayo et al. (2023) noted no impact on 75 g/kg of APP inclusion, suggesting a need to enhance the feed value of APP with any antinutrient ameliorating process to enable higher APP inclusion in Jumbo quail diets. The liver and spleen are vital organs involved in various physiological functions such as metabolism, detoxification, and immune response (Yang et al. 2021). Thus, the absence of dietary effects on liver and spleen sizes suggests that the addition of APP in the Jumbo quail diet did not cause any significant damage, inflammation, or abnormal growth in these organs. The inclusion of non-conventional feed ingredients in poultry diets can directly influence meat quality by altering its physical attributes, nutritional composition, and chemical properties, which can impact consumer preferences (Mazizi et al. 2020). Meat lightness, redness, and chroma measured 24 h post-mortem exhibited linear or quadratic effects in response to increasing APP levels.

The presence of phenols with antioxidant properties in APP (Table 2) impacts meat colour by interacting with pigments and enzymes involved in colour formation (Efenberger-Szmechtyk et al. 2021). Consequently, this affects the stability and degradation of myoglobin, which could have been responsible for the noted changes in meat lightness, redness, and chroma. The pH values of meat play a critical role in determining its quality during storage and processing. Meat pH is a crucial factor for meat quality because it impacts attributes such as taste, WHC, tenderness, shelf life, and juiciness (Muchenje et al. 2009). The lack of treatment effects on WHC, drip loss, and cooking loss may be linked to the similarities in meat pH among the treatment groups, signifying that the inclusion of APP does not affect meat juiciness. Additionally, the similar shear force values indicate that dietary APP inclusion has no effect on meat tenderness.

## Conclusion

The incorporation of apple pomace powder had negative impacts on performance metrics of young growing Jumbo quail. However, the inclusion of 50–75 g APP levels /kg diet maximized feed efficiency and final body weight in five-week-old Jumbo quail without adversely affecting blood and meat quality parameters.

## Data Availability

The data that support the findings of this study are available on request from the corresponding author.
